# Effect of dietary soybean oil and antioxidants on fatty acids and volatile compounds of tail subcutaneous and perirenal fat tissues in fattening lambs

**DOI:** 10.1186/s40104-016-0083-y

**Published:** 2016-04-12

**Authors:** Yongjia Peng, Jiakun Wang, Jia Lin, Jianxin Liu

**Affiliations:** Laboratory of Ruminant Nutrition, College of Animal Sciences, Zhejiang University, 866 Yuhangtang Road, Hangzhou, 310058 Zhejiang P. R. China; College of Biological, Chemical Science and Engineering, Jiaxing University, 118 Jiahang Road, 314001 Jiaxing, Zhejiang P. R. China

**Keywords:** Aldehydes, Flavor, Oxidation, Unsaturated fatty acids

## Abstract

**Background:**

Fat is the primary source of the volatiles that determine the characteristic flavors of animal products. Because unsaturated fatty acids (UFAs) contribute to changes in flavor as a result of the oxidation process, a feeding trial was performed to investigate the effects of dietary soybean oil or antioxidants on the fatty acid and volatile profiles of the tail subcutaneous (SF) and perirenal fat tissues (PF) of fattening lambs. Thirty-six Huzhou lambs were assigned to four dietary treatments in a randomized block design. The lambs’ diets were supplemented with soybean oil (0 or 3 % of DM) or antioxidants (0 or 0.025 % of DM).

**Results:**

Neither soybean oil nor antioxidant supplementation had an effect on lamb growth (*P* > 0.05). In regard to tail SF, soybean oil supplementation increased the 18:2n6t (*P* < 0.05) and the total amount of volatile acids, whereas antioxidant supplementation increased the content of C18:2n6c and C18:3n3 (*P* < 0.05) but had no effect on the volatiles profile. In regard to PF, dietary soybean oil supplementation increased the C18:0 content (*P* < 0.01); decreased the C18:1 (*P* = 0.01), C22:1 n9 (*P* < 0.01) and total UFA (*P* = 0.03) contents; and tended to decrease the E-2-octenal (*P* = 0.08), E, E-2, 4-decadienal (*P* = 0.10), 2-undecenal (*P* = 0.14) and ethyl 9-decenoate (*P* = 0.10) contents. Antioxidant supplementation did not affect either the fatty acid content or the volatiles profile in the PF.

**Conclusions:**

Tail SF and PF responded to dietary soybean oil and antioxidant supplementation in different ways. For SF, both soybean oil and antioxidant supplementation increased the levels of unsaturated fatty acids but triggered only a slight change in volatiles. For PF, soybean oil supplementation decreased the levels of unsaturated fatty acids and oxidative volatiles, but supplementation with antioxidants had little effect on PF fatty acids and the volatiles profile.

## Background

The isomerization and hydrolysis effects of ruminal microbial enzymes result in ruminant-derived products containing higher n-3 polyunsaturated fatty acids (PUFA) and conjugated linoleic acids, which have been shown to benefit human health. Thus, dietary supplementation with PUFA-rich vegetable oil, fish oil or oil seeds is an effective strategy for increasing PUFA levels in meat or milk products [1–3]; for instance, several studies have reported increased C18:2 and C18:3 levels in lamb and goat meat in response to soybean oil supplementation [4, 5]. At the same time, however, higher levels of PUFAs in animal products may alter the flavor of the meat. Study results have been inconclusive and often contradictory, with some researchers suggesting that higher PUFA concentrations in muscle tissues might result in reduced meat quality [6, 7], whereas others have noted that higher proportions of C18:3 n3 in lamb phospholipids are associated with reductions in abnormalities in lamb flavor [8]. Because PUFAs are very sensitive to oxidization, the inconsistent results could be attributed to the various intermediate products of oxidation of different PUFAs [9], such as E,E-2,4-decadienal, an oxidant product of linoleic acid (C18:2) and the source of “oil” aroma, which contributes to the change in flavor of the cooked meat of lambs whose diet was supplemented with sunflower oil [10]. Many studies have focused on protecting PUFAs from oxidation through the use of antioxidants, and several synthetic antioxidants, such as butylated hydroxy anisole (BHA), butylated hydroxy toluene (BHT) and alpha tocopherol have been successfully employed to prevent or restrict lipid oxidation in meat products [11].

Fat tissues are the source of many valuable products in the food industry. For example, sheep store excess fat in their tails during times of abundant food, and this tail fat is used to produce ghee, a type of clarified butter [12]. Perirenal fat along with the triceps brachii muscles can be used to produce hamburger meat [13]. Given that the generation of flavor volatiles is highly dependent on the cooking method, most studies have focused on the flavor development of cooked meat, but there is scant information about raw meat. The fatty acids and volatiles in raw animal tissues could be considered as the basal components that play a part in the complex reactions between fatty acids and other non-volatiles during cooking; it is therefore desirable to identify the fatty acids and volatiles in fat tissue, as the solvents of volatiles. Because the effect of dietary soybean oil supplementation on the volatiles profile in the raw tissue of lambs is limited, we hypothesized that dietary soybean oil supplementation (3 % DM) might increase the level of PUFAs in tail subcutaneous and perirenal fat tissues of fattening lambs, with coinciding antioxidant supplementation to minimize PUFA oxidation in fat tissues.

Huzhou sheep, renowned for their rapid growth rates and high fertility, are among the most common breed of sheep raised in China. Here, we examined the effects of dietary supplementation with a UFA (soybean oil) and antioxidants on the fatty acid and volatiles profile of the tail SF and PF of fattening Huzhou lambs.

## Methods

### Animals and management

The experimental procedures used here, including the feeding, transport and slaughter of the subject sheep, were approved by the Zhejiang University Experimental Animal Welfare Ethics Committee.

Thirty-six 7-month-old male Huzhou male lambs (29.9 kg ± 2.2 kg [mean ± SD]) were randomly divided into four groups based on a randomized block design, with each group composed of three units of three lambs. Four dietary treatments (concentrate:forage ratio of 5:5) categorized by soybean oil and antioxidant as the main effects (Tables 1 and 2) were used, with treatments consisting of 1) basal diet without supplementation (C); 2) basal diet supplemented with antioxidants (0.025 % DM of Agrado Plus, a proprietary blend of antioxidants that includes ethoxyquin and silicon dioxide; Novus International Inc., St. Charles, MO, USA), designated as the Antioxidant group (A); 3) basal diet supplemented with soybean oil (3 % DM), designated as the Oil group (O); and 4) basal diet supplemented with both soybean oil and antioxidants, designated as the Oil and Antioxidant group (OA). All groups were fed equal portions twice daily at 0830 and 1630 h, and the lambs were given free access to drinking water. Feeding trials were conducted for a period of 7 wks, consisting of 1 wk for adaptation followed by 6 wks of treatment. Feed intake and residual food amounts were recorded throughout the testing period.

**Table 1:** Ingredients and chemical composition of the diet^a^ (%, DM basis)

Items	Diets^b^			
	C^c^	A^d^	O^e^	AO^f^
Ingredients, % as DM basis				
Peanut vine	50.0	50.0	50.0	50.0
Corn	23.7	23.7	0.0	0.0
Wheat bran	2.8	2.8	28.4	28.4
Rapeseed cake	8.3	8.3	3.4	3.4
Tofu dreg	13.1	13.1	13.1	13.1
Soybean oil	0.0	0.0	3.0	3.0
Antioxidant	0.0	0.025	0.0	0.025
Salt	0.8	0.8	0.8	0.8
CaHPO_4_	0.5	0.5	0.5	0.5
NaHCO_3_	0.3	0.3	0.3	0.3
Premix^g^	0.5	0.5	0.5	0.5
Chemical composition				
DM, %	79.8	79.8	80.4	80.4
DE, MJ/kg	12.9	12.9	13.0	13.0
CP, % of DM	15.4	15.4	15.3	15.3
Ca, % of DM	1.6	1.6	1.5	1.5
P, % of DM	0.4	0.4	0.5	0.5

^a^Diet was formulated to meet the Feeding Standards of Meat-producing Sheep and Goats (Ministry of Agriculture of P.R. China, 2004)

^b^Diets included four treatments (C, A, O and OA) and are the same as in Tables 2, 3, 4, 5, 6 and Fig. 1

^c^C is the control group; the diet did not contain antioxidants or soybean oil

^d^A is the antioxidant group; the diet consisted of the control diet plus antioxidant (0.025 % of DM)

^e^O is the soybean oil group; the diet consisted of the control diet plus soybean oil (3 % of DM), and the dietary energy and protein levels were adjusted to match those of the control diet

^f^OA is the soybean oil plus antioxidant group; the diet consisted of the soybean oil diet plus antioxidant (0.025 % of DM)

^g^Formulated to provide (per kilogram of DM) 1 200 000 IU of vitamin A, 280 000 IU of vitamin D, 5 000 mg of vitamin E, 14 000 mg of Zn, 3 500 mg of Mn, 3 000 mg of Cu, 200 mg of I, 60 mg of Co and 100 mg of Se

**Table 2:** Fatty acid composition of the diet (percentage of total fatty acids)

Fatty acids, %	C/A	O/OA
C10:0	0.04	0.01
C12:0	0.34	0.19
C14:0	0.32	0.20
C15:0	0.03	0.02
C16:0	8.26	7.93
C16:1	0.17	0.11
C17:0	0.12	0.10
C18:0	3.39	3.15
C18:1 n9c	14.26	11.38
C18:2 n6t	0.20	0.35
C18:2 n6c	17.64	22.23
C18:3 n3	2.48	2.81
C20:1	0.72	0.31
C20:5 n3	0.13	0.07
C22:1 n9	1.68	0.86
C23:0	0.06	0.12
C24:0	0.04	0.06
C22:6 n3	0.02	0.01
C24:1 n9t	0.10	0.10
Saturated	12.60	11.77
Unsaturated	37.40	38.23

### Sample collection

At the end of the experiment, all lambs were weighed prior to the morning feeding for two consecutive days and transported to a slaughterhouse after being fasted for 24 h. The total PF and right side of the tail fat were sliced following removal of the vessels and connective tissues, and approximately 20 g of the PF and tail SF were subsampled and vacuum-packed after slaughter. The samples were stored at 4 °C for 24 h, followed by storage at −80 °C for the subsequent determination of volatiles and fatty acids.

### Fatty acids analysis

Fatty acid methyl esters (FAMEs) were produced from 20 mg of fat samples via the one-step trans-esterification method, in accordance with the procedures described by Rule [14]. The FAMEs were dissolved in 0.9 mL of hexane and 0.1 mL of methyl heneicosanoate as an internal standard (1 mg/mL) and then transferred to clean vials for gas chromatography (GC) analysis according to the procedures described in a previous study [15]. In brief, 20-mg fat samples were placed in 10-mL screw-capped tubes, to which 1 mL each of a boron trifluoride methanol solution and methanol were added. The tubes were then placed in an 80 °C water bath for 2 h and vortexed every 5 min. After the tubes had cooled, 1.5 mL of hexane and 1.5 mL of double distilled water were added and thoroughly mixed. Upon cooling to room temperature, 1 mL of the upper layer was transferred to a new tube and dried by nitrogen. The FAMEs were dissolved in 0.9 mL of hexane and 0.1 mL of methyl heneicosanoate (1 mg/mL) and then transferred to clean vials prior to GC analysis.

A GC 6890 N with an FID detector (Agilent Technologies Inc., CA, USA) equipped with a DB-23 column (30 m long, 0.25 mm ID, 0.25-μm film) (Agilent Technologies Inc., CA, USA) was used to analyze the fatty acid profiles of the samples at injector and detector temperatures of 220 °C and 260 °C, respectively. The temperature program consisted of an initial temperature of 70 °C, an increase at a rate of 58 °C/min to 240 °C and a final temperature of 240 °C for 5 min. Fatty acids were identified by comparison to known external standard mixes of 37 FAMEs (Sigma Aldrich, China). Methyl-heneicosanoate was selected as the internal standard, with the quantity of each fatty acid calculated according to the relative peak area of the internal standard.

### Volatile compounds analysis

Headspace solid phase micro-extraction (SPME) coupled with gas chromatography-mass spectrometry (GC-MS) was used to analyze the volatiles content of fat tissue, as described elsewhere [15]. Briefly, SPME with 50/30 mm divinylbenzene/carboxen/polydimethylsiloxane fiber was used to extract the volatiles from 1-g samples of fat tissues at 120 °C. A DB-5 capillary column (30 m × 0.25 mm × 0.25 mm) (Agilent Technologies Inc., CA, USA) was used to analyze the volatiles. After desorption of SPME at 250 °C for 5 min, volatiles were separated under the following chromatographic conditions: GC oven temperatures were increased from 40 to 250 °C at a rate of 38 °C/min and then held at 250 °C for 5 min, with helium used as the carrier gas at a flow rate of 0.8 mL/min. The electron impact energy was set at 70 eV, and data were collected in the range of m/z 40–650. The Wiley library and mass spectral database (NIST 2002, Washington, DC, USA) coupled to the Kovats retention indices taken from a series of standards (C6-C25 n-alkanes) were used to identify the mass spectra of the volatile compounds.

### Statistical analysis

Growth performance, fatty acid content and volatiles profile data were analyzed using the GLM procedure of the SAS software system (version 9.1). The model included soybean oil, antioxidants and the interaction between soybean oil and antioxidants. The means were compared when the interaction terms of the model were significant (*P* < 0.05) using the LAMEANS and PDIFF separation of the entire group. Discriminant function analysis (DFA) was performed to distinguish the characteristics of the volatiles among the four groups. All data were normalized with a log10 transformation prior to DFA.

## Results

### Growth performance

As shown in Table 3, no significant effect of soybean oil and antioxidant on growth performance was detected, but final body weight (*P* = 0.13) and average daily gain (ADG) (*P* = 0.08) were slightly reduced in sheep undergoing the soybean oil treatment. Antioxidant supplementation tended to decrease dry matter intake (DMI) (*P* = 0.10), final body weight (*P* = 0.07) and the ADG of lambs (*P* = 0.07).

**Table 3:** Effects of supplementation with soybean oil, antioxidant or soybean oil plus antioxidant on growth of fattening lambs

Items	Diet				SEM	*P*-value		
	C	A	O	AO		O^a^	A^b^	O × A^c^
Number of lambs	9	9	9	9				
Initial body weight, kg	29.8	30.0	30.0	29.7	0.58			
Final body weight, kg	37.5	35.6	35.8	35.1	0.61	0.13	0.07	0.39
Dry matter intake, g/d	1213	1137	1160	1078	42.0	0.22	0.10	0.95
Average daily gain, g/d	188	147	148	141	11.3	0.08	0.07	0.16

^a^The effect of soybean oil, the same as in Tables 4, 5 and 6

^b^The effect of antioxidant, the same as in Tables 4, 5 and 6

^c^The interactive effect of soybean oil and antioxidant, the same as in Tables 4, 5 and 6

### Fatty acid profile

The primary effects of soybean oil and antioxidant supplementation on the fatty acid profiles of SF and PF are shown in Table 4. Palmitic acid (16:0), oleic acid (18:1) and stearic acid (18:0) were the three major fatty acids in both SF and PF, accounting for more than 85 % of the total fatty acid content.

**Table 4:** Effects of supplementation with soybean oil, antioxidant or soybean oil plus antioxidant on fatty acid composition of subcutaneous and perirenal fat tissue in fattening lambs

Fatty acids, g/100 g FAME	Diet				RMSE^a^	*P*-value		
	C	A	O	AO		O	A	O × A
Subcutaneous fat tissue								
Total FA^b^	209	211	213	199	44.92	0.79	0.73	0.60
C10:0	0.34	0.34	0.31	0.36	0.15	0.94	0.65	0.64
C12:0	0.39	0.32	0.42	0.37	0.15	0.47	0.26	0.88
C14:0	4.64	4.15	4.81	4.47	0.88	0.41	0.18	0.81
C14:1	0.81	0.93	0.75	0.95	0.60	0.92	0.44	0.86
C15:0	1.08	1.22	1.08	1.19	0.29	0.87	0.21	0.88
C16:0	25.3	24.0	25.8	25.1	2.13	0.29	0.18	0.71
C16:1	2.98	2.60	2.93	2.81	0.97	0.81	0.47	0.69
C17:0	1.58	1.86	1.46	1.65	0.31	0.13	0.03	0.66
C18:0	14.8	14.8	13.8	13.3	4.15	0.40	0.86	0.85
C18:1	43.0	44.0	43.2	44.0	2.82	0.90	0.36	0.97
C18:2 n6t	0.89	1.00	1.13	1.04	0.18	0.03	0.93	0.11
C18:2 n6c	3.74	4.20	3.75	4.17	0.65	0.97	0.06	0.93
C18:3 n6	0.11	0.11	0.13	0.08	0.07	0.94	0.37	0.35
C18:3 n3	0.41	0.51	0.40	0.51	0.12	0.90	0.02	0.95
UFA	66.7	68.1	66.1	66.9	3.05	0.40	0.31	0.76
U/S	2.03	2.16	1.96	2.05	0.26	0.33	0.24	0.84
Perirenal fat tissue								
Total FA^b^	145	114	105	119	28.47	0.09	0.41	0.03
C10:0	0.23	0.21	0.20	0.17	0.07	0.16	0.31	0.97
C12:0	0.31	0.33	0.33	0.42	0.29	0.59	0.62	0.72
C14:0	3.32	3.11	2.99	2.68	0.64	0.10	0.25	0.82
C15:0	0.84	0.84	0.86	0.84	0.11	0.89	0.90	0.75
C16:0	24.5	23.0	23.9	22.4	2.49	0.53	0.11	1.00
C17:0	1.64	1.78	1.51	1.55	0.13	0.00	0.07	0.28
C18:0	36.2	37.3	39.2	43.3	4.14	0.00	0.09	0.30
C18:1	27.4	27.9	25.8	23.6	3.06	0.01	0.42	0.21
C18:2 n6c	3.70	3.92	3.82	3.55	1.05	0.73	0.95	0.50
C18:3 n6	0.18	0.17	0.18	0.17	0.05	0.88	0.50	0.88
C18:3 n3	0.53	0.45	0.48	0.44	0.19	0.62	0.34	0.74
C20:0	0.52	0.53	0.49	0.58	0.14	0.88	0.28	0.44
C20:1	0.36	0.28	0.23	0.22	0.15	0.10	0.43	0.52
C22:1 n9	0.28	0.21	0.07	0.14	0.09	0.00	0.96	0.03
UFA	32.5	32.9	30.6	28.1	4.22	0.03	0.49	0.32
U/S	0.48	0.50	0.44	0.40	0.09	0.02	0.57	0.31

^a^ *RMSE* root mean square error, the same as in Tables 5 and 6

^b^The amount of total FA is expressed as mg/g fat tissue

For SF, soybean oil supplementation only increased the content of C18:2 n6t (*P* = 0.03), whereas antioxidant supplementation increased the contents of C17:0 (*P* = 0.03), C18:3 n3 (*P* = 0.02) and C18:2 n6c (*P* = 0.06). No fatty acid was affected by the interaction of soybean oil and antioxidant.

For PF, soybean oil supplementation increased the content of C18:0 (*P* < 0.01) and decreased the proportion of total UFA (*P* = 0.03), which was mainly attributed to decreases in C18:1 (*P* = 0.01) and C22:1 n9 contents (P < 0.01). Antioxidant supplementation did not affect the fatty acid composition of PF (*P* > 0.05). The interaction between soybean oil and antioxidant significantly affected the total amount of FA (*P* = 0.03) and the C22:1n9 content of the PF (*P* = 0.03).

### Volatile compounds profile

A total of 35 volatile compounds were identified in SF and PF and classified according to their chemical nature as acids, aldehydes, alcohols, esters and others (Tables 5 and 6). Aldehydes and esters were the two major types of volatile compounds in both fat tissues, accounting for approximately 70 % of the total volatiles detected.

**Table 5:** Effects of supplementation with soybean oil, antioxidant or soybean oil plus antioxidant on volatile profiles in subcutaneous fat tissues of fattening lambs

Component	Abb.^a^	RI^b^	CSID^c^	Diet				RMSE	*P*-value		
				C	A	O	AO		O	A	O*A
Aldehydes				36.5	36.4	35.0	32.6	14.50	0.61	0.81	0.82
Hexanal	Ad1	798	5949	-	0.7	1.9	-		-	-	-
E-2-Heptenal	Ad2	955	4446437	1.3	1.2	1.1	0.8	1.06	0.41	0.62	0.82
Phenylacetaldehyde	Ad3	1040	13876539	2.1	2.1	2.0	1.6	1.35	0.49	0.68	0.64
E-2-Octenal	Ad4	1056	4446445	1.6	2.0	1.4	3.4	2.99	0.57	0.29	0.47
Nonanal	Ad5	1104	29029	12.1	13.5	14.3	7.2	8.07	0.48	0.33	0.14
E-2-Nonenal	Ad6	1157	4446456	12.3	11.1	8.0	8.6	6.45	0.15	0.92	0.70
E,E-2,4-Decadienal	Ad7	1295	4446470	4.1	3.4	4.0	4.1	2.26	0.67	0.72	0.60
2-Undecenal	Ad8	1368	4446477	3.0	2.5	2.3	7.0	6.46	0.42	0.38	0.27
Esters				34.4	39.1	39.8	42.5	14.36	0.40	0.48	0.84
Ethyl octanoate	Es1	1193	7511	1.8	1.1	1.0	4.7	4.35	0.36	0.34	0.16
Methyl decanoate	Es2	1328	7759	3.0	1.1	1.3	5.0	5.70	0.59	0.67	0.17
Ethyl cyclohexanepropanoate	Es3	1345	55387	5.1	9.8	6.5	5.0	7.00	0.50	0.54	0.22
Methyl 2,8-dimethyldecanoate	Es4	1353	487217	3.0	2.9	1.1	1.1	2.17	0.02	0.90	0.96
Ethyl 9-decenoate	Es5	1389	455568	3.2	2.0	2.8	1.8	1.91	0.64	0.12	0.89
Ethyl caprinate	Es6	1398	7757	2.1	5.0	12.0	7.4	10.33	0.11	0.83	0.31
Methyl 2,4,6-trimethylundecanoate	Es7	1487	487035	0.3	0.3	0.5	0.5	0.34	0.14	0.78	0.85
Methyl undecanoate	Es8	1490	14847	2.0	2.6	1.5	1.9	1.57	0.28	0.42	0.85
Ethyl 9-oxononanoate	Es9	1537	17861	-	3.6	-	-		-	-	-
Methyl laurate	Es10	1554	7847	0.8	0.7	0.7	0.8	0.48	0.81	0.78	0.58
Ethyl laurate	Es11	1597	7512	3.3	1.2	1.9	2.4	2.87	0.92	0.43	0.20
Geranyl isovalerate	Es12	1606	4515295	0.8	1.3	0.7	0.8	0.79	0.27	0.23	0.51
Methyl 2,6-dimethyltridecanoate	Es13	1651	487205	1.1	2.1	2.0	1.6	1.70	0.70	0.67	0.28
Methyl myristate	Es14	1769	29024	2.2	1.8	3.5	2.4	2.97	0.38	0.45	0.73
Ethyl myristate	Es15	1793	29023	5.6	3.6	4.3	7.1	7.68	0.69	0.89	0.39
Acids				8.1	10.7	12.4	14.7	5.11	0.03	0.20	0.92
(2E)-2-Methyl-2-nonenoic acid	Ac1	1269	4724999	1.7	1.4	2.8	2.1	2.20	0.28	0.52	0.79
Decanoic acid	Ac2	1355	2863	2.2	3.0	4.5	6.1	4.00	0.07	0.42	0.76
Undecanoic acid	Ac3	1465	7888	0.5	0.7	0.8	0.9	0.48	0.15	0.23	0.74
Lauric acid	Ac4	1537	3756	-	1.5	1.4	1.7		-	-	-
Tridecylic acid	Ac5	1621	12013	0.7	2.0	1.2	0.9	1.49	0.57	0.33	0.17
Alcohols				12.8	9.0	8.9	8.1	6.22	0.29	0.30	0.50
Heptan-1-ol	Al1	969	7837	4.0	3.0	3.8	2.7	3.91	0.86	0.47	0.94
1-Octanol	Al2	1069	932	1.1	1.1	1.1	1.1	0.96	0.91	0.99	0.89
2-Methyl-1-dodecanol	Al3	1492	38544	4.4	2.0	2.2	2.8	3.87	0.62	0.50	0.28
2-Hexyl-1-decanol	Al4	1790	86034	3.4	2.9	1.8	1.5	1.78	0.03	0.55	0.96
Others				8.2	4.8	3.8	2.2	3.50	0.01	0.06	0.46
Toluene	Ot1	762	1108	3.7	0.9	1.4	-		-	-	-
2-Pentadecanone	Ot2	1696	55242	4.7	3.8	2.4	2.1	2.42	0.03	0.48	0.71

^a^All volatile compounds were grouped according to chemical categories. Ad, Ac, Al, Es and Ot are abbreviations for the aldehyde, acid, alcohol, ester and “other” groups, respectively, the same as in Table 6

^b^RI, retention indices of individual compounds relative to C6-C25 n-alkanes, the same as in Table 6

^c^CSID, ChemSpider ID of each chemical (http://www.chemspider.com/), the same as in Table 6

**Table 6:** Effects of supplementation with soybean oil, antioxidant or soybean oil plus antioxidant on volatile profiles in perirenal fat tissues of fattening lambs

Component	Abb.	RI	CSID	Diet				RMSE	*P*-value		
				C	A	O	AO		O	A	O*A
Aldehydes				34.3	41.4	38.6	31.7	8.65	0.37	0.98	0.03
Hexanal	Ad1	798	5949	1.6	-	4.2	2.6				
E-2-Heptenal	Ad2	955	4446437	2.0	2.2	2.4	1.6	2.02	0.90	0.68	0.53
Phenylacetaldehyde	Ad3	1040	13876539	1.6	1.6	6.6	0.3	5.08	0.31	0.09	0.08
E-2-Octenal	Ad4	1056	4446445	1.5	1.9	0.5	0.7	1.52	0.08	0.61	0.83
Nonanal	Ad5	1104	29029	3.6	4.5	4.8	4.9	2.79	0.45	0.66	0.68
E-2-Nonenal	Ad6	1157	4446456	2.1	4.3	2.4	1.9	2.85	0.31	0.42	0.19
E,E-2,4-Decadienal	Ad7	1295	4446470	19.5	24.7	16.3	18.1	8.98	0.10	0.29	0.59
2-Undecenal	Ad8	1368	4446477	3.0	3.6	1.4	2.3	2.72	0.14	0.46	0.86
Esters				34.8	33.9	34.9	41.9	8.14	0.17	0.31	0.17
Ethyl octanoate	Es1	1193	7511	3.6	3.0	4.6	4.2	3.05	0.34	0.68	0.89
Methyl decanoate	Es2	1328	7759	10.5	6.1	7.6	10.5	5.74	0.73	0.75	0.10
Ethyl cyclohexanepropanoate	Es3	1345	55387	1.8	2.1	1.8	1.5	1.47	0.61	0.92	0.55
Methyl 2,8-dimethyldecanoate	Es4	1353	487217	1.5	2.5	1.4	2.3	2.56	0.87	0.32	0.95
Ethyl 9-decenoate	Es5	1389	455568	2.1	2.3	1.8	1.2	1.13	0.10	0.63	0.26
Ethyl caprinate	Es6	1398	7757	3.4	5.1	2.8	5.4	5.95	0.95	0.31	0.83
Methyl 2,4,6-trimethylundecanoate	Es7	1487	487035	0.3	0.6	0.7	1.8	1.10	0.06	0.13	0.33
Methyl undecanoate	Es8	1490	14847	2.5	2.7	2.5	3.4	2.68	0.73	0.60	0.72
Ethyl 9-oxononanoate	Es9	1537	17861	2.3	-	3.1	6.6				
Methyl laurate	Es10	1554	7847	0.8	1.0	3.4	1.5	2.21	0.13	0.37	0.27
Ethyl laurate	Es11	1597	7512	1.6	2.5	1.1	2.2	1.77	0.56	0.16	0.81
Geranyl isovalerate	Es12	1606	4515295	0.7	1.4	0.8	0.8	0.81	0.32	0.23	0.23
Methyl 2,6-dimethyltridecanoate	Es13	1651	487205	0.6	0.8	2.2	0.6	1.30	0.20	0.22	0.08
Methyl myristate	Es14	1769	29024	1.4	3.0	0.9	1.9	3.10	0.44	0.25	0.76
Ethyl myristate	Es15	1793	29023	1.8	3.5	3.0	2.5	3.88	0.94	0.70	0.42
Acids				16.0	12.0	11.1	14.3	11.15	0.74	0.92	0.36
(2E)-2-Methyl-2-nonenoic acid	Ac1	1269	4724999	4.1	4.8	3.8	3.5	2.14	0.34	0.81	0.52
Decanoic acid	Ac2	1355	2863	-	-	1.8	1.5				
Undecanoic acid	Ac3	1465	7888	9.6	1.9	1.1	3.1	11.71	0.40	0.51	0.25
Tridecylic acid	Ac5	1621	12013	3.1	1.7	3.1	1.9	1.94	0.85	0.08	0.93
(7Z)-7-Tetradecenoic acid	Ac7	1777	4471826	0.7	1.5	0.9	0.9	0.67	0.41	0.13	0.08
Alcohols				7.9	8.7	10.1	6.8	3.69	0.92	0.35	0.11
Heptan-1-ol	Al1	969	7837	1.5	1.2	2.1	0.8	1.12	0.80	0.06	0.26
1-Octanol	Al2	1069	932	3.3	3.1	3.0	2.1	1.82	0.40	0.45	0.63
2-Methyl-1-dodecanol	Al3	1492	38544	1.8	2.9	3.3	3.4	2.85	0.34	0.54	0.61
2-Hexyl-1-decanol	Al4	1790	86034	1.5	2.1	2.5	1.3	2.25	0.96	0.72	0.24
Others				7.0	4.0	5.3	5.5	3.26	0.92	0.23	0.18
Toluene	Ot1	762	1108	0.8	1.1	-	1.2				
2-Pentadecanone	Ot2	1696	55242	6.2	3.3	5.3	4.3	3.37	0.95	0.12	0.42

As shown in Table 5, dietary soybean oil supplementation increased the content of total acids (*P* = 0.03) and decreased the contents of methyl 2,8-dimethyldecanoate, 2-hexyl-1-decanol and 2-pentadecanone in SF (*P* < 0.05); moreover, soybean oil supplementation led to slightly decreased E-2-nonenal (*P* = 0.11) levels, and increased ethyl caprinate, decanoic acid and undecanoic acid (0.05 < *P* < 0.20) levels. No volatile compounds were affected by antioxidant treatment or by the interaction between soybean oil and antioxidant.

As shown in Table 6, levels of E-2-octenal, E,E-2,4-decadienal, 2-undecenal and ethyl 9-decenoate tended to decrease in response to soybean oil supplementation (0.05 < *P* < 0.20), but no volatile compounds were affected by the antioxidant treatment. The total content of aldehydes was affected by the interaction between soybean oil and antioxidant supplementation (*P* = 0.03).

**Fig. 1:**
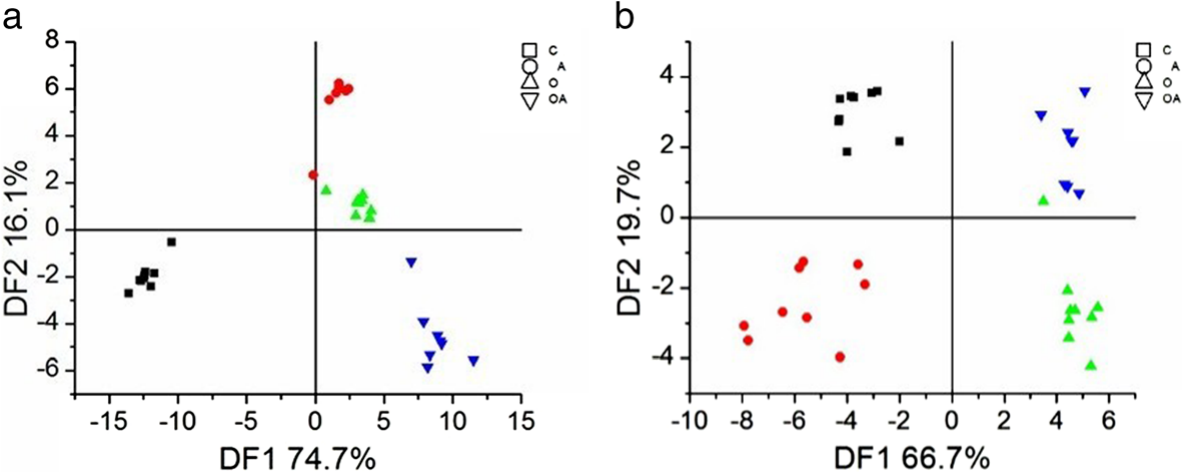
DFA plots of volatile profiles of subcutaneous (**a**) and perirenal (**b**) fat tissues from lambs fed normal diets (■, C), diets supplemented with soybean oil (▲, O), diets supplemented with antioxidant (●, A) and diets supplemented with soybean oil plus antioxidant (▼, AO)

All of the volatile compounds detected in SF and PF were subjected to discriminant function analysis (DFA) (Fig. 1). The DFA plot based on the volatiles profile of SF is shown in Fig. 1a. In DF1 (74.7 %), the C group was distinguished from the other three groups (A, O and OA groups), but those groups were not separated from one another; however, the O group was separated from the OA group in DF2 (16.1 %). The DFA plot based on the volatiles profile of PF is shown in Fig. 1b. In DF1 (66.7 %), the C and CA groups were separated from the O and OA groups, but the C group was not distinguished from the CA group, and the O group was not separated from the OA group. In DF2 (19.7 %), the C group was separated from the CA group, and the O group was separated from the OA group.

## Discussion

### Growth performance

To maintain equal energy and protein levels between the control and soybean oil-supplemented diets, a higher percentage of wheat bran was used instead of corn in the soybean oil diet, which might increase the satiety of lambs in groups O and OA and thus reduce their DMI and final body weights. Moreover, the effects of dietary soybean oil supplementation on ruminant growth performance were not consistent. Based on our findings both here and in a previous study of Huzhou lambs, soybean oil supplementation did not influence the growth performance of finishing lambs [16, 17]; on the contrary, several studies have reported significantly negative effects of dietary soybean oil on the growth performances of both steers and lambs and suggested that the UFA in the soybean oil may impair rumen fermentation and fiber digestibility [18, 19]. Potential negative effects of UFAs on rumen fermentation should therefore be of concern.

Contrary to what we expected, dietary antioxidant supplementation tended to negatively affect lamb growth. Agrado Plus is a commercial antioxidant used in feed, and the results of several studies – including our own previous research – have demonstrated its beneficial effect on the health and performance of dairy cattle [20, 21]. Here, the reasons for the negative effects of antioxidant supplementation on lamb growth were undetermined; it may simply be due to differences in the physiologies of sheep and dairy cattle.

### Fatty acid profile

Similar to the increased C18:2 in tail SF observed here, dietary PUFA-rich soybean oil supplementation improved the content of C18:2 in the intramuscular fat of goats and lambs [4, 19]. Because C18:2 is the main fatty acid in soybean oil, the increased proportion of C18:2 in the SF may be due to the dietary C18:2 that was not subjected to biohydrogenation in the rumen. In our previous study of dairy cattle, dietary antioxidants counteracted the negative effects of dietary low saturated fats (mainly C18:1) and increased C18:1 levels in the milk [21], which suggested that antioxidant supplementation had a positive influence on UFA accumulation. In this study, however, antioxidant supplementation increased the concentrations of both C18:2 and C18:3 in SF regardless of whether it was ingested as part of a normal diet or a diet enriched with soybean oil, providing a positive signal that the use of antioxidants might improve the nutritional value of Huzhou lamb tail SF.

Differences between internal (perirenal) and external (subcutaneous) fat deposits have been widely demonstrated. In this study, more UFAs were detected in SF, whereas more SFAs were detected in PF, accounting for 70 % of the total fatty acids in PF. This finding is consistent with the higher SFA concentrations previously observed in internal (kidney) fat compared with external fat depots [22]. As Lee et al. [23] reported, stearoyl-CoA desaturase (SCD) activity was higher in SF than in PF, which partially explains the higher SFA proportion observed in the PF in this study. The fatty acid profile in PF changed in a different manner than did that of SF in response to dietary supplementation regardless of whether the supplement was soybean oil or antioxidant, similar to observations made by Lee et al. [24], who supplemented the diet of lambs with ground whole-fat soybeans. Moreover, Berthelot et al. showed that the differential uptake of FA from the rumen contributes to variations in trans-fatty acid proportions in the PF, SF and muscles in response to vitamin E supplementation [25].

### Volatile compounds profile

Volatile components are not necessarily odor-active. As reviewed by Watkins et al., only 15 of 187 volatiles were identified as the primary components of lamb aroma based on a gas chromatography − olfactometry (GC-O) analysis, including the aldehydes E,E-2,4-decadienal, Z-2-nonenal, E-2-heptenal, methional, E-2-nonenal, decanal, 2,4-E,E-heptadienal, octanal and E-2-octenal [10]. Meanwhile, one indicator, termed the odor-activity value, was calculated and used to represent the contribution of volatiles to food flavor [26]. Bueno et al. built a partial least-squares model based on the odor-activity value of 32 volatiles and concluded that alkenals and alkadienals have negative effects on the intensity of lamb flavor and that E,E-2,4-decadienal and E-2-nonenal were the most abundant volatiles [27]. We found similar patterns in this study: the main aldehydes in SF (such as nonanal, E-2-nonenal and E,E-2,4-decadienal) and those in PF (E,E-2,4-decadienal) largely determine the flavor characteristics of SF and PF.

When soybean oil was added to the lambs’ diet, the slight decrease in E-2-nonenal (*P* = 0.15) observed in the SF was inconsistent with the increase in C18:2, as E-2-nonenal is the oxidative product of C18:2, suggesting that the extent of oxidation in SF might be lower than what we assumed, but the exact reasons for this phenomenon remain unknown. Moreover, the addition of soybean oil tended to increase the content of the volatile decanoic acid (*P* = 0.07). The odor of decanoic acid is reported to be positively associated with the oxidation of wine, contributing to the “animal”, “bitterness” and “dairy” characteristics of wine [28]. Enhanced decanoic acid content would therefore suggest an increase in SF bitterness as a result of soybean oil supplementation.

In regard to PF, given that the odor threshold of E-2-octenal is only “4” – that is, the flavor of E-2-octenal becomes recognizable at concentrations above 4 ng/g tissue – and despite the content of E-2-octenal decreasing by 1 and 1.2 % with soybean oil supplementation (C vs O: 1.5 % vs 0.5 %; A vs AO: 1.9 % vs 0.7 %), the flavor of the PF still became less “green, nutty and fatty”, descriptors that depict the typical flavor of E-2-octenal. Moreover, E,E-2,4-decadienal (with a typical flavor described as “fatty and fried foods”) was the primary aldehyde found in PF, but its concentration decreased in response to dietary soybean oil supplementation, suggesting that the intensity of “fatty” or “fried”-like flavors of PF was more subdued.

Compared with the effects of soybean oil supplementation, antioxidant supplementation triggered fewer changes in both SF and PF. In SF, although antioxidant supplementation led to higher concentrations of C18:2 and C18:3, the fact that we did not detect a simultaneous increase in the oxidative by-products (aldehydes) of these UFAs is an indication that antioxidant supplementation may improve anti-oxidative performance and thus hinder the progress of UFA oxidation. In the PF, the interaction effect between soybean oil and antioxidant supplementation on aldehydes suggested that the presence of the antioxidant slows the rate of accumulation of oxidative by-products. Thus, although the antioxidant did not induce any direct flavor-related changes in the composition of the volatiles, it may suppress UFA oxidation in fat tissues and thus have an indirect positive effect on meat flavor.

To visually represent the different responses of SF and PF to dietary soybean oil and antioxidant supplementation, given the complexity of the factors that determine the flavor of fat, the DFA plots of the volatile contents provide an intuitive outline of the differences between each sample. From Fig. 1a, it can be seen that soybean oil supplementation might change the flavor of SF when no antioxidants are added (74.7 %, C vs O), but less so when the antioxidant is added (16.1 %, A vs AO); antioxidant supplementation, meanwhile, induced large differences in the absence of soybean oil (74.7 %, C vs A) but did not trigger obvious changes when delivered in conjunction with soybean oil supplementation (16.1 %, O vs OA). Thus, the effect of soybean oil supplementation on SF flavor was dependent on whether or not the antioxidant was present. As seen in ure 1b, soybean oil supplementation led to clear changes when the antioxidant was added (66.7 %, C/CA vs O/OA) and the antioxidant alone also altered the volatiles composition (21.9 %, C vs CA, O vs OA), but the extent of change caused by the latter scenario was less than that of soybean oil supplementation. We can therefore infer that dietary soybean oil supplementation had an effect on PF flavor independent of the presence or absence of the antioxidant.

## Conclusions

In summary, dietary soybean oil supplementation improved the UFA content in tail SF, and antioxidant supplementation further enhanced UFAs by suppressing the accumulation of oxidative volatiles, thus interacting with the effect of soybean oil on SF flavor discrimination. Dietary soybean oil supplementation induced an decrease in the levels of saturated fatty acids and aldehydes in PF. Antioxidant supplementation, however, had little effect on the fatty acid and volatiles composition in the PF.

## Abbreviations

ADG: average daily gain
BHA: butylated hydroxy anisole
BHT: butylated hydroxy toluene
CLA: conjugated linoleic acid
DFA: discriminant function analysis
DMI: dry matter intake
FAMEs: fatty acid methyl esters
GC: gas chromatography
GC-MS: gas chromatography-mass spectrometry
GC-O: gas chromatography − olfactometry
PF: perirenal fat tissue
PUFA: polyunsaturated fatty acids
SF: subcutaneous fat tissue
SFA: saturated fatty acids
SPME: solid phase micro-extraction
UFA: unsaturated fatty acid
